# Glutathione Deficit Affects the Integrity and Function of the Fimbria/Fornix and Anterior Commissure in Mice: Relevance for Schizophrenia

**DOI:** 10.1093/ijnp/pyv110

**Published:** 2015-10-03

**Authors:** Alberto Corcoba, Pascal Steullet, João M. N. Duarte, Yohan Van de Looij, Aline Monin, Michel Cuenod, Rolf Gruetter, Kim Q. Do

**Affiliations:** Laboratory for Functional and Metabolic Imaging, École Polytechnique Fédérale de Lausanne, Lausanne, Switzerland (Mr Corcoba, and Drs Duarte, Van de Looij, and Gruetter); Center for Psychiatric Neuroscience, Department of Psychiatry, Lausanne University Hospital, CHUV, Lausanne, Switzerland (Mr Corcoba, Drs Steullet, Monin, Cuenod, and Do); Division of Child Growth & Development, University of Geneva, Geneva, Switzerland (Dr Van de Looij); Department of Radiology, University Hospital, Lausanne, Switzerland (Dr Gruetter); Department of Radiology, University Hospital, Geneva, Switzerland (Dr Gruetter).

**Keywords:** Schizophrenia, anterior commissure, fimbria-fornix, oxidative stress, glutathione

## Abstract

**Background:**

Structural anomalies of white matter are found in various brain regions of patients with schizophrenia and bipolar and other psychiatric disorders, but the causes at the cellular and molecular levels remain unclear. Oxidative stress and redox dysregulation have been proposed to play a role in the pathophysiology of several psychiatric conditions, but their anatomical and functional consequences are poorly understood. The aim of this study was to investigate white matter throughout the brain in a preclinical model of redox dysregulation.

**Methods:**

In a mouse model with impaired glutathione synthesis (Gclm KO), a state-of-the-art multimodal magnetic resonance protocol at high field (14.1 T) was used to assess longitudinally the white matter structure, prefrontal neurochemical profile, and ventricular volume. Electrophysiological recordings in the abnormal white matter tracts identified by diffusion tensor imaging were performed to characterize the functional consequences of fractional anisotropy alterations.

**Results:**

Structural alterations observed at peri-pubertal age and adulthood in Gclm KO mice were restricted to the anterior commissure and fornix-fimbria. Reduced fractional anisotropy in the anterior commissure (-7.5%±1.9, *P*<.01) and fornix-fimbria (-4.5%±1.3, *P*<.05) were accompanied by reduced conduction velocity in fast-conducting fibers of the posterior limb of the anterior commissure (-14.3%±5.1, *P<.*05) and slow-conducting fibers of the fornix-fimbria (-8.6%±2.6, *P<.*05). Ventricular enlargement was found at peri-puberty (+25%±8 *P<.*05) but not in adult Gclm KO mice.

**Conclusions:**

Glutathione deficit in Gclm KO mice affects ventricular size and the integrity of the fornix-fimbria and anterior commissure. This suggests that redox dysregulation could contribute during neurodevelopment to the impaired white matter and ventricle enlargement observed in schizophrenia and other psychiatric disorders.

## Introduction

Abnormal redox homeostasis and oxidative stress have been proposed to play a role in the etiology of several psychiatric disorders. In recent years, a convincing body of evidence has been gathered for bipolar and anxiety disorders, depression, autism, and specially schizophrenia (see comprehensive reviews in Ng et al., 2008; Smaga et al., 2015). Oxidative stress and altered antioxidant systems have been considered a hallmark of schizophrenia at least in subgroups of patients (Do et al., 2009; Yao and Keshavan, 2011; Flatow et al., 2013; Fournier et al., 2014). Altered expression of genes related to oxidative stress (Middleton et al., 2002; Prabakaran et al., 2004), oxidative damage to lipids (Wang et al., 2009) and nucleic acids (Che et al., 2010), as well as reduced glutathione levels in the nervous tissue of patients (Do et al., 2000; Yao and Leonard, 2006; Gawryluk et al., 2011) could contribute to the pathology, but may as well be a consequence of years of disability and/or neuroleptic medication (Szabó et al., 1983; Middleton et al., 2002). In this regard, increased lipid peroxidation, altered activity of antioxidant enzymes, and decreased glutathione levels in the plasma of drug-naive patients (Mahadik et al., 1998; Raffa et al., 2009) suggest that impaired redox homeostasis is not a consequence of chronicity. Impaired glutathione synthesis (Tosic et al., 2006; Gysin et al., 2007) and metabolism (Gravina et al., 2010; Rodríguez-Santiago et al., 2010) in schizophrenia may have a genetic origin. Moreover, dysfunction of proteins coded by other risk genes, including Disc1 (Park et al., 2010; Johnson et al., 2013), Dysbindin (Gokhale et al., 2012), Neuregulin (Goldshmit et al., 2001), and hypo-function of NMDA receptors (Papadia et al., 2008; Baxter et al., 2015), have been shown to affect the antioxidant systems and/or cause oxidative stress.

Magnetic resonance (MR) imaging (MRI), and especially diffusion tensor imaging (DTI), have provided a powerful tool to noninvasively study white matter (WM) and thus facilitated the study of the anatomical basis of brain connectivity in vivo. Structural anomalies of WM have been reported in several psychiatric disorders, including schizophrenia, bipolar disorder, depression, and autism (Thomason and Thompson, 2011; Shizukuishi et al., 2013), with affected areas often overlapping between different conditions (White et al., 2008b). This suggests that similar pathological mechanisms may be at the origin of these alterations. Schizophrenia in particular is thought to result from a dysfunctional integration or disconnection within the brain (Friston and Frith, 1995). Anomalies in WM structure have been reported at both chronic (Ellison-Wright and Bullmore, 2009) and early stages of the disorder (Samartzis et al., 2013; Yao et al., 2013). Reduced myelin content measured by other myelin-sensitive MRI techniques (Flynn et al., 2003; Du et al., 2013) suggests that deficient myelination may underlie these abnormalities. This hypothesis is supported by the reduced number of oligodendrocytes (Hof et al., 2002; Byne et al., 2006; Vostrikov et al., 2007), abnormal myelin ultra-structure (Miyakawa et al., 1972; Uranova et al., 2001), and altered myelin-related gene expression (Hakak et al., 2001; Tkachev et al., 2003; Roussos et al., 2012) found in postmortem brains of patients.

Based on the susceptibility of oligodendrocytes to oxidative stress (Back et al., 1998) and on the regulation of their proliferation and differentiation by the intracellular redox state (Smith et al., 2000; French et al., 2009) and glutathione levels (Monin et al., 2014), we hypothesize that redox dysregulation could contribute to WM pathology in schizophrenia, the disorder in which both have been more frequently reported, but possibly also in other neurodevelopmental conditions. This hypothesis is supported by our observations that prepubertal mice with low glutathione levels (Gclm KO mice, which carry a functional deletion in the modulatory subunit of the glutamate-cysteine ligase, the key enzyme in the synthesis of glutathione) display delayed oligodendrocyte maturation and myelination in the anterior cingulate cortex (Monin et al., 2014). Accumulation of N-acetylaspartate in the prefrontal cortex (Duarte et al., 2011) of these mice suggests myelination deficits, since N-acetylaspartate is required by oligodendrocytes to synthesize myelin (Chakraborty et al., 2001; Kirmani et al., 2002). Moreover, in young human adults, we have found a positive correlation between the glutathione levels in the anterior cingulate cortex and the fractional anisotropy (FA) along the cingulum (Monin et al., 2014).

The present study assesses the impact of a glutathione deficit on WM integrity in a longitudinal MRI study of the brain of Gclm KO mice from peri-puberty to full adulthood. Using cutting-edge multimodal MR at high magnetic field, we investigated WM structure throughout the brain with DTI, the neurochemical profile in the anterior cortex with MR spectroscopy (MRS) and the ventricular volume with T2-weighted MRI, as enlargement of the ventricles is a morphological hallmark of schizophrenia pathology but is also found in other psychiatric diseases such as bipolar disorder and autism (Arnone et al., 2009; Lange et al., 2015). Further functional evaluation of WM tracts displaying abnormal DTI parameters was performed with standard electrophysiological methods. Such reverse translational approach will help to understand the functional significance of the anomalies in DTI-derived parameters found in patients and screen for underlying pathological mechanisms.

## Methods

### Animals

Mice (Yang et al., 2002) were bred and kept in a temperature- and humidity-controlled facility under a 12-h-light/-dark cycle with free access to food and water. All experiments were approved by the local veterinary authority.

### MRS and MRI

All data were acquired on a 14.1 T horizontal-bore magnet (Magnex Scientific, Abingdon, UK) equipped with a 12-cm inner diameter gradient (400 mT/m in 200 µs) and interfaced with a DirectDrive console (Agilent Technologies, Palo Alto, CA). Radio frequency transmission and reception was achieved with a home-built quadrature surface coil of 18mm diameter with a geometry adapted to the mouse brain. Male Gclm KO (n=15) and WT (n=16) mice were scanned longitudinally at postnatal days (PNDs) 40, 97, and 180. Each scanning session consisted of MRS, DTI, and anatomical MRI acquisitions amounting to 4 hours total scanning time per animal. Mice were anesthetized with 1% to 1.5% isoflurane in 1:1 air:O2 mixture and fixed in a home-built holder. Body temperature was maintained at 37.0±0.5°C by warm water circulation. The mouse brain was positioned in the iso-center of the magnet, and field homogeneity was achieved before each acquisition with FAST(EST)MAP (Gruetter and Tkác, 2000). MRS acquisition and analysis were performed as detailed before (Duarte et al., 2011) in a volume of interest (0.9×4×1.6mm^3^) placed in the prefrontal cortex. To ensure reproducible position within the scanner across animals, a sagittal image was acquired to place the top of the rhinal fissure at +4mm of the iso-center in the bore longitudinal direction. The center of the MRS voxel was then placed at +2mm of the iso-center, so the voxel edges were at 2.8 and 1.2mm from the rhinal fissure, respectively; Bregma +0.76 and +2.36 coordinates in the mouse brain atlas (Paxinos and Franklin, 2001). In the other 2 axes, the voxel was centered manually in the midline, and its bottom edge was aligned to the top of the corpus callosum.

Diffusion-weighted images were acquired using a pulse field gradient sequence with TR=2 seconds, TE=31.35 milliseconds, field-of-view=20x20mm, matrix size =128x64, slice thickness=0.6mm, number of slices=15, number of averages=4, 6 diffusion directions (-1,1,0; 0,1,1; -1,0,1; -1,-1,0; 0,-1,1; 1,0,1; and their opposites to cancel b-value cross terms; Neeman et al., 1991) and b-value=1000mm^2^/s. Anatomical scans were acquired using a fast spin-echo sequence (TR = 2 seconds, TE = 43.36 milliseconds, Echo Train Length = 8, field-of-view = 20 x 20mm, matrix size = 256 x 256, slice thickness = 0.6 m, number of slices = 15, number of averages = 20). To ensure reproducibility of the area imaged across animals, the first slice of the DTI and anatomical images was centered at the top of the rhinal fissure.

### MRI Analysis

A tensor was fitted to each voxel in the diffusion-weighted images using FSL Dtifit (Behrens et al., 2003), and maps of FA and radial, axial, and mean diffusivities (RD, AD, MD) were derived. Cohort MRI studies require registration of images to a common space of reference to enable the analysis of corresponding spatial localizations across subjects. As registration target, we used a home-built atlas of the C57Bl/6 mouse brain segmented in 27 regions of interest (ROI) (supplementary Figure 1). The anatomical scan from each animal was registered to the atlas using FSL FLIRT and FNIRT (Jenkinson and Smith, 2001; Jenkinson et al., 2002; Andersson et al., 2007), and the inverse of the transformations was used to transport the segmented atlas into each animal’s space. FA, MD, RD, and AD maps were then multiplied by the back-transformed binary masks to obtain average values within each ROI for each animal. Ventricular volume was assessed by manual segmentation of the T2-weighted anatomical images using FSLview (all FSL programs were provided by FMRIB’S Software Tools, Oxford, UK).

### Electrophysiology

Adult mice (90±10 days of age) were used for the electrophysiological experiments. Recordings were performed on horizontal brain slices in which fibers from the corpus callosum (CC), fimbria-fornix (FF), and both anterior and posterior limbs of the anterior commissure (AC) run longitudinal to the slicing plane. The CC was studied within the same slice as the FF; this corresponded to the posterior part of the genu of the CC. Experiments were performed at 28°C to slow down conduction velocity and thus allow better separation between the action potentials generated by fast-conducting and slow-conducting fibers. The precise distance between the stimulation electrode (a bipolar tungsten electrode placed within the WM tract) and the recording glass electrode was measured with a calibrated ocular micrometer. The recording electrode was lowered within the WM tract until the compound action potential (CAP), induced by a 100-µs bipolar voltage pulse, reached maximum amplitude. Stimulations evoked 2 distinct CAPs, associated respectively with fast-conducting and slow-conducting fibers (supplementary Figure 2). The relationships between the stimulus intensity and CAP amplitude, the conduction velocity, and the refractory period for the fast- and slow-conducting fibers of each WM tract were measured (see supplementary Figure 3 for methods regarding evaluation of the refractory period).

### Statistical Analyses

Statistical analyses were performed in R (R Foundation for Statistical Computing, 2012). Spectroscopy data were analyzed by fitting a mixed-effects linear model to the concentration of each metabolite using genotype, age, and their interaction as fixed effects. As random effect, we used intercepts for each subject. *P*-values were obtained from likelihood ratio tests of a full-model against a model without the effect in question. Significant genotype-age interactions were analyzed posthoc with unpaired *t* tests between genotypes at each age applying Holm correction for the 3 comparisons. The same methods were used to analyze each DTI-derived diffusivity parameters (FA, RD, AD, MD) applying Holm correction for the 27 ROIs. Visual inspection of residual plots revealed no deviation from normality. Autocorrelation functions were fitted to all repeated measurements data to assess independence between ages. F-ratio tests were performed to assess homoscedasticity.

A mixed-effects linear model was also fitted to the volumetric data as described above, but we detected heteroscedasticity between genotypes. Thus, we analyzed the effect of age within each genotype with a 1-way ANOVA and the effect of genotype with 3 unpaired *t* tests (at each age independently with Welch approximation to correct for heteroscedasticity).

Conduction velocity and refractory period were compared between genotypes using independent *t* tests, and the relationship between stimulus intensity and CAP amplitude (normalized or absolute) was compared between genotypes using a Generalized Additive Mixed Model with a random effect at the individual level to take into account the interdependence of the responses to different stimulus intensities within a fiber tract of the same individual.

## Results

**Figure 1.**
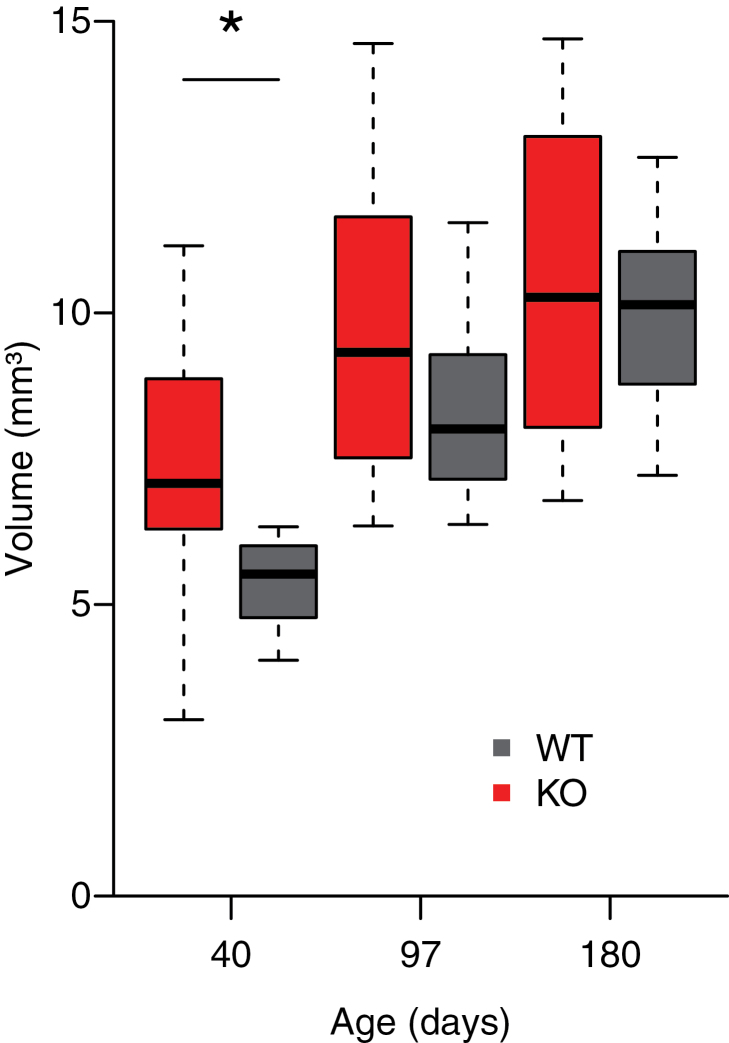
Ventricular volume in Gclm KO and wild-type (WT) mice along development. Each box plot depicts group average (horizontal black lines), inter-quartile range (box), and 95% confidence interval (whiskers). *P<.05 significant effect of genotype, unpaired 2-tailed *t* test with Welch approximation to correct for heteroscedasticity.

To determine major structural changes, we measured ventricular volume using T2-weighted MRI. Ventricular volume displayed higher variability in KO than in WT mice (F-ratio=2.9, *P*=.04) (Figure 1). An increase in ventricular size with age was confirmed by analyzing separately the data from WT (F1,28=98.2, *P<.*0001) and KO mice (F1,30=26.9, *P<.*0001). T tests between genotypes at each age (correcting for the unequal variances) revealed significantly larger ventricular volume in KO compared with WT mice at PND 40 (+25.0%±8.1, *P*=.02), a trend at PND 97 (+17.0%±6.4, *P*=.05), but not at PND 180 (+6.7%±7.4, *P*=.5).

**Figure 2.**
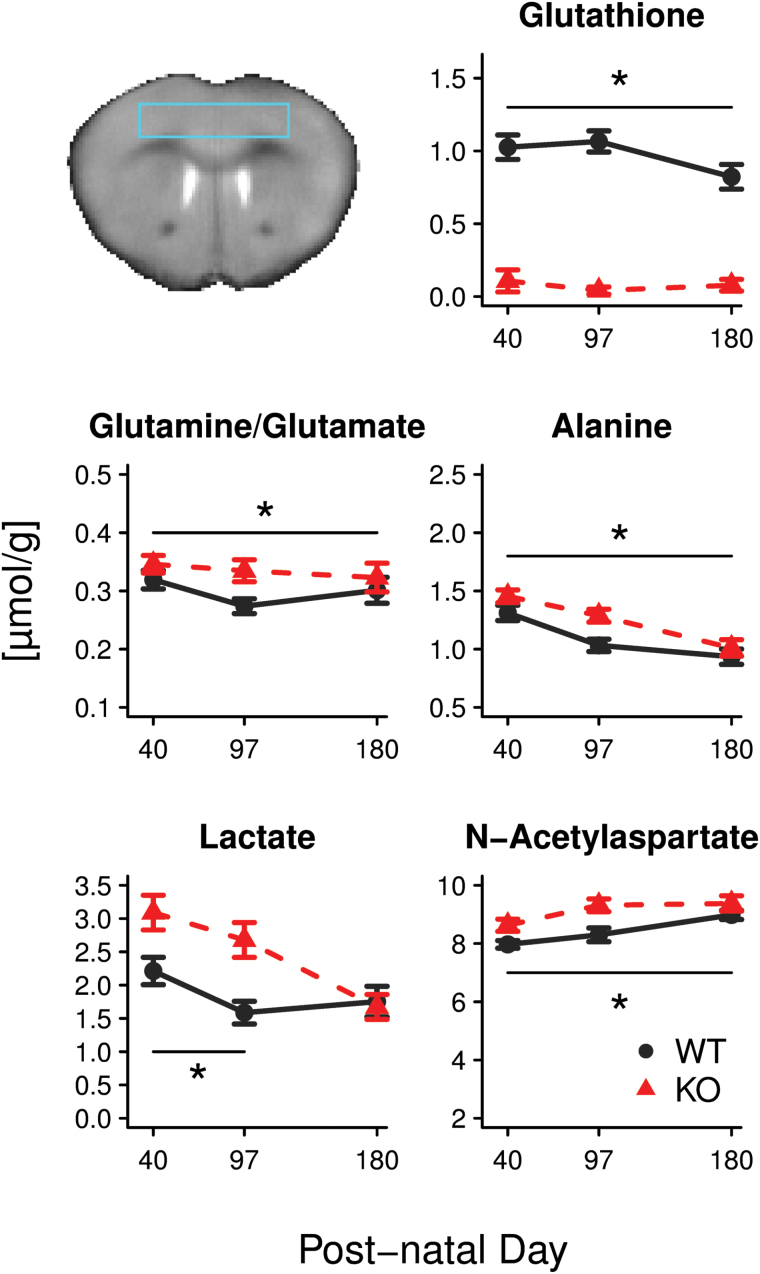
Neurochemical alterations in the cortex of Gclm KO mice relative to controls (out of the whole neurochemical profile analyzed). Mean±SEM are shown. *P<.05, significant genotype effect. Top left panel shows the position of the voxel used for the acquisition.

To validate and extend previous findings observed during development in Gclm KO mice (Duarte et al., 2011), we assessed the concentrations of 20 metabolites in the prefrontal cortex of KO and WT mice at peri-puberty and young and full adulthood (Figure 2). Significant differences between genotypes were found for glutathione (-84.7%±5.1 in KO, *P<.*0001), N-acetylaspartate (+8.7%±2.3, *P*=.0005), glutamine/glutamate ratio (+11.8%±5.3, *P*=.03), and alanine (+11.3%±3.4, *P*=.003). Lactate showed a significant interaction between genotype and age (*P*=.01), and posthoc *t* tests showed a significant difference between genotypes at PND 40 (+39.8%±6.1 in KO, *P*=.042) and 97 (+68.9%±8.9, *P*=.002) but not at 180 (-4.6%±6.8, *P*=.79).

**Figure 3.**
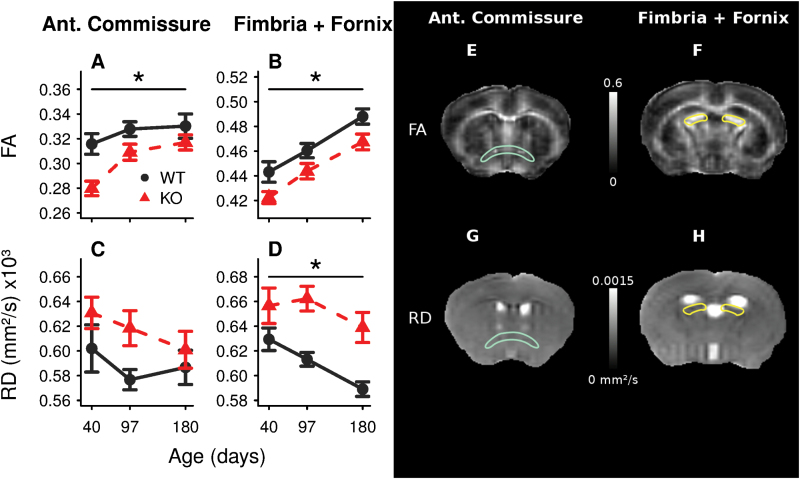
Anomalies in diffusivity parameters derived from diffusion tensor imaging (DTI) in Gclm KO mice. Fractional anisotropy (FA) in the anterior commissure (AC) (A) and fimbria-fornix (FF) (B) and radial diffusivity (RD) in the AC (C) and FF (D) along the development of Gclm KO and wild-type (WT) mice. The graphs depict group average±SEM. *P<.05 for genotype effect corrected for multiple comparisons (see Methods). The right panel presents FA (E-F) and RD (G-H) images from a representative animal. Highlighted for spatial reference are the AC in blue (E,G) and the FF in yellow (F,H).

To determine anomalies in WM integrity, we analyzed DTI-derived diffusivity parameters in 27 ROIs throughout the brain of Gclm KO and WT mice at PND 40, 97, and 180. We found no significant interaction of genotype with age in any of the diffusivity parameters or ROIs analyzed. A significant increase of FA with age in 22 ROIs was accompanied by a decrease of RD in 6 of them. Between genotypes, lower FA in the AC (one region comprising both the anterior and posterior limbs, -7.5%±1.9, *P*=.005) and FF (-4.5%±1.3, *P*=.03) of KO relative to WT mice attained statistical significance after Holm correction for multiple testing (Table 1, Figure 3). Of all other parameters analyzed (RD, AD, and MD; supplementary Tables 1–3), only an increase of RD in the FF was detected in KO compared with WT mice (+6.6%±1.7, *P*=.01) (Figure 3).

**Table 1.** Mean FA Values Per ROI and Genotype, Difference between Genotypes, and Standard Error of the Difference Estimated by a Mixed-Effects Linear Model Using Genotype and Age as Fixed Factors and Intercepts for Each Subject as Random Factor

Region of interest	Mean WT	Mean KO	WT-KO (%)	WT-KO SD (%)	p	p corrected
Fornix and Fimbria	0.43	0.41	4.5	1.3	0.001	*0.034
Internal Capsule and Pallidum	0.41	0.40	1.6	0.8	0.054	1
Dorsal Hippocampal Commissure	0.40	0.39	3.3	1.5	0.023	0.629
Medulla and Pons	0.38	0.36	5.4	3.1	0.08	1
Corpus Callosum	0.37	0.37	0.7	1.6	0.641	1
Anterior Commissure	0.31	0.28	7.5	1.9	0	*0.005
Septum	0.29	0.31	-4.2	1.4	0.004	0.096
Cingulum	0.29	0.29	-2.6	1.2	0.028	0.746
Midbrain	0.28	0.28	-0.9	1.7	0.587	1
External Capsule	0.27	0.26	1.7	1.1	0.122	1
Thalamus	0.24	0.24	0.7	1.0	0.496	1
Basal Ganglia	0.24	0.24	-1.8	1.9	0.317	1
Superior Colliculi	0.23	0.23	0.4	2.1	0.846	1
Subiculum	0.23	0.24	-5.0	3.5	0.147	1
Hypothalamus	0.23	0.22	3.8	2.3	0.098	1
Amygdala and Amygdaloid	0.23	0.22	0.8	2.8	0.766	1
Olfactory Nucleus	0.22	0.23	-4.9	2.0	0.018	0.476
Dorsal Raphe	0.21	0.22	-3.9	3.3	0.233	1
Entorhinal, Piriform and Insular Cortex	0.21	0.21	-0.9	1.3	0.485	1
Orbital Cortex	0.20	0.21	-3.8	1.5	0.011	0.308
Periaqueductal Gray	0.20	0.21	-5.6	2.3	0.013	0.356
Frontal Association and Motor Cortex	0.18	0.18	-1.8	1.2	0.127	1
Caudate and Putamen	0.17	0.18	-2.4	1.5	0.098	1
Prelimbic and Cingulate Cortex	0.17	0.17	-0.8	1.2	0.501	1
Sensory and Visual Cortex	0.16	0.17	-4.5	2.0	0.03	0.806
Dorsal Hippocampus	0.16	0.16	-0.4	2.0	0.847	1
Ventral Hippocampus	0.14	0.14	-2.1	3.3	0.526	1

The first and second columns are calculated by averaging the values of the 3 ages for each animal and then the average of all animals of the respective genotype. The third column is calculated as (mean WT – mean KO) x 100/mean WT. The fourth column shows the SD of the difference (mean WT – mean KO) also in percentage of mean WT. *P*-values for genotype differences from the likelihood ratio tests before and after correction for multiple comparisons are given in the fifth and sixth columns, respectively.

**Figure 4.**
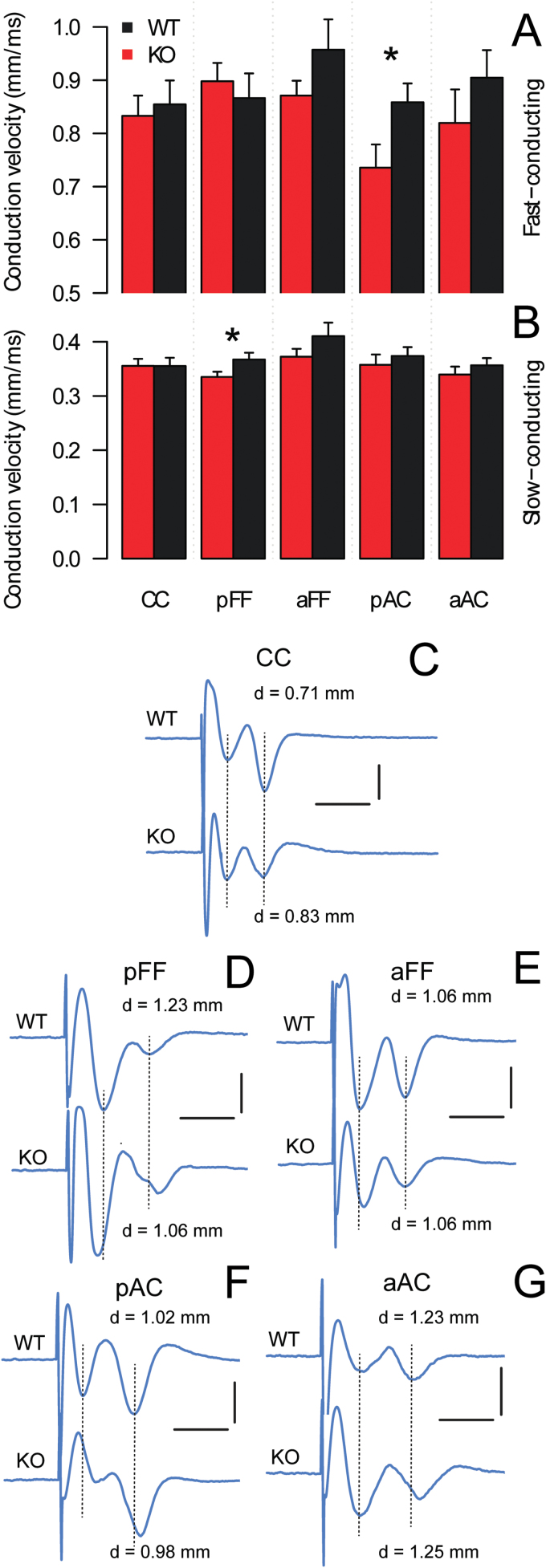
Conduction velocity along the fast- (A) and slow-conducting fibers (B) in the corpus callosum (CC), the fornix-fimbria (FF), and the anterior commissure (AC) of adult Gclm KO and wild-type (WT) mice. Mean±SEM are shown (n=8–11/group). *P<.05 unpaired 1-tailed *t* test. Traces (C-G) are representative recordings of compound action potentials (CAPs) evoked in each of these fiber tracts in Gclm KO and WT mice. Horizontal bars: 2ms; vertical bars: 1 mV; d is the measured distance between the stimulating and recording electrodes. pFF, posterior part of FF; aFF, anterior part of FF; aAC, anterior limb of AC; pAC posterior limb of AC.

To assess the functional consequences of the structural anomalies detected in the DTI experiment, we performed electrophysiological recordings within the FF and AC and within the CC as a negative control. The anterior and posterior limbs of the AC were assessed separately, while insufficient resolution did not allow this in the DTI experiment. In each WM tract, electrical stimulation evoked 2 distinct CAPs, associated respectively with fast- and slow-conducting fibers (supplementary Figure 2). However, the CAP amplitude from the slow-conducting fibers was much smaller (even absent in 2 of 11 cases) in the posterior than in the anterior part of the FF (supplementary Figure 2). Therefore, separate recordings were systematically performed within both parts of the FF. We found a small but significant decrease in the conduction velocity along the fast-conducting fibers in the posterior limb (-14.3%±5.1, *P*=.024) of the AC and the slow-conducting fibers of the posterior part of the FF (-8.6%±2.6, *P*=.027) in KO compared with WT mice (Figure 4), but there was no alteration in the CC. The refractory period was not affected in either fast- or slow-conducting fibers of the AC or FF in Gclm KO mice (supplementary Figure 3). The relationship between stimulus intensity and absolute CAP amplitude was not different between genotypes in any WM tracts (supplementary Figure 4). However, when the CAP amplitude was normalized to the maximal response to render the response-dose independent of the number and density of excited fibers, the relationship between stimulus intensity and normalized CAP amplitude was significantly different between genotypes for the fast-conducting fibers of the posterior limb (*P*=.046) and the slow-conducting fibers of the anterior limb of the AC (*P*=.004) (supplementary Figure 5). These results suggest alterations of physical properties (such as axonal diameter) in these fibers. Together, these data reveal subtle alterations within FF and AC but not CC of KO mice, thus confirming a functional outcome of the structural anomalies detected by DTI.

## Discussion

The present longitudinal multimodal MR study at 14.1 T and follow-up electrophysiological experiments demonstrated neurochemical, structural, and functional anomalies in Gclm KO mice, a model of redox dysregulation relevant to schizophrenia and other psychiatric disorders. These mice displayed small but significant alterations in WM structure of the FF and AC as assessed by DTI from peri-puberty onwards. Reduced conduction velocity along both tracts was consistent with FA alterations. Ventricular enlargement and neurochemical profile anomalies were also found in peri-pubertal Gclm KO mice, but some tended to normalize at full adulthood. This highlights the critical role of the redox system during the development of brain structures known to be affected in psychiatric disorders such as schizophrenia. Interestingly, a parallel DTI study in early psychosis patients revealed similar alterations in the FF (P. S. Baumann, A. Griffa, M. Fournier, C. Ferrari, L. Alameda, M. Cuenod, J.P. Thiran, P. Hagmann, K.Q. Do, and P. Conus, unpublished observations).

### Enlarged Ventricles

Gclm KO mice displayed ventricular enlargement at peri-pubertal age and young adulthood, but not later. Ventricular enlargement is a neuroanatomical hallmark of schizophrenia pathology (Wright et al., 2000; Shenton et al., 2001) present already in first-episode patients (Steen et al., 2006; Vita et al., 2006). Likewise, both bipolar adolescents and adults with psychotic symptoms show enlarged ventricles (Edmiston et al., 2011). Interestingly, Buckman and collaborators (1987) found a strong negative correlation between glutathione peroxidase activity in blood cells and ventricular volume in schizophrenia patients. These data suggest a link between oxidative stress and ventricular enlargement, but they could also be associated with psychotic symptoms, since decreased glutathione peroxidase activity in blood cells is found during acute relapse and in chronic inpatients but not in stable outpatients or during first-episode psychosis (Flatow et al., 2013). Our data suggest that redox imbalance caused by a glutathione deficit could be implicated in the ventricular enlargement during development, but not necessarily in adulthood as observed in bipolar (Strakowski et al., 2002), autistic (Lange et al., 2015), and chronic schizophrenia (Kempton et al., 2010) patients. The Gclm- KO model addresses only one risk factor, the genetic one. Thus, the ventricle enlargement anomaly could be compensated in adulthood in the absence of additional genetic and/or environmental risks, as is likely the case in patients.

### Altered Neurochemical Profile

Our MRS results pointed to altered metabolism in the cortex of Gclm KO mice. They confirmed that many alterations observed previously in young Gclm KO mice (Duarte et al., 2011) were still present in young adults, though some tended to normalize later. The increase in glutamine/glutamate ratio, lactate, and N-acetylaspartate concentrations suggest alterations in the glutamatergic neurotransmission system (Duarte et al., 2011) and in the metabolic coupling between neurons and glia (for review, see Tranberg et al., 2004; Yang et al., 2014). The elevated N-acetylaspartate level is of particular interest in the context of the present study, since N-acetylaspartate synthesized in neurons is used by oligodendrocytes for myelin production (Moffett et al., 2013). Although N-acetylaspartate accumulation may suggest an impairment of axon-glia interactions, this remains speculative, as N-acetylaspartate concentration cannot be measured in vivo in the same WM tracts.

### Structural and Functional WM Anomalies

DTI quantifies water displacement within a tissue to probe micro-structure, especially in WM where water movement is constrained by the myelin sheaths wrapping the axons. In WM, both de- and dys-myelination cause an increase of radial diffusivity–the magnitude of diffusion perpendicular to the main tract orientation–and a reduction in diffusion anisotropy (Song et al., 2002; Harsan et al., 2006; Ou et al., 2009; Ruest et al., 2011). Although DTI measurements are altered along many WM tracts in schizophrenia, depression, anxiety, and bipolar and other disorders (Thomason and Thompson, 2011; Shizukuishi et al., 2013), the underlying causes of these structural anomalies and their functional consequences remain unclear. We demonstrated that the modest but significant FA decrease within the FF and AC of Gclm KO mice is associated with reduced conduction velocity. By contrast, DTI parameters and conduction velocity appeared normal in the CC. The mechanism by which the conduction velocity is decreased in the AC and FF is currently unknown. In both the anterior and posterior limbs of the AC of KO mice, the conduction velocity was or tended to be lower in the fast- (putatively myelinated) but not slow-conducting fibers (putatively not or less myelinated). This suggests anomalies at the level of the myelin sheath and/or the interplay between oligodendrocytes and axons (Ritter et al., 2013). In the FF of KO mice, however, the reduced FA may be in part independent of oligodendrocytes, since the conduction velocity was affected in slow-conducting fibers, which likely represent non- or weakly myelinated axons. It is counter-intuitive, though, that a radial diffusivity increase in Gclm KO mice–which is typically considered a sign of myelin alteration–attained statistical significance in the FF. In the AC, in contrast, this was only true before the stringent correction for multiple comparisons was applied. Together, our data highlight that even a modest alteration of DTI parameters in a WM tract can be associated with a small reduction of axonal conduction not exclusively restricted to the myelinated fibers.

### Vulnerability of the FF and AC to Redox Dysregulation

Our data revealed that the FF and AC are vulnerable to a deficit in glutathione. Based on DTI parameters, both WM tracts are already affected in peri-pubertal KO mice and remain so through adulthood. Moreover, the axonal conductivity is also reduced along these 2 tracts, which could potentially impair proper communication between the brain regions directly connected via these fiber tracts, particularly when very precise temporal coherent activity is critical. The FF connects the hippocampus with the hypothalamus and other subcortical areas, including the septal nuclei, nucleus accumbens, mammillary bodies, and thalamic anterior nucleus. These structures play a role in a range of behaviors impaired in schizophrenia, such as memory retrieval, emotionality, or motivation (White et al., 2008a). Reduced anisotropy in the FF has been reported in both chronic (Kuroki et al., 2006; Ellison-Wright and Bullmore, 2009; Fitzsimmons et al., 2009; Qiu et al., 2010) and first-episode patients (Luck et al., 2010; Fitzsimmons et al., 2014; Lee et al., 2013). This suggests that anomalies in the FF appear early in the illness. Likewise, Gclm KO mice showed reduced FA in the FF at peri-pubertal age and adulthood. In a complementary study, we found that early psychosis patients displayed decreased FA along the fornix and reduced hippocampal volume, which are associated with peripheral markers of redox homeostasis (P. S. Baumann, A. Griffa, M. Fournier, C. Ferrari, L. Alameda, M. Cuenod, J.P. Thiran, P. Hagmann, K.Q. Do, and P. Conus, unpublished observations). Noteworthy, adult Gclm KO mice have also reduced number of parvalbumin-immunoreactive interneurons and impaired gamma oscillations in the ventral hippocampus (Steullet et al., 2010). Therefore, the structural and functional alterations in the FF may be linked to hippocampal anomalies. MRI studies have also unveiled reduced FA in the fornix (Barysheva et al., 2013) and reduced volume of the fimbria (Elvsåshagen et al., 2013) in bipolar patients and reduced FA in the fornix in children with autism (Poustka et al., 2012), suggesting common pathological mechanisms probably comprising oxidative stress.

The AC is comprised by 2 independent bundles: the anterior and posterior limbs. The anterior limb allows inter-hemispheric communication between the 2 contra-lateral olfactory systems (Kucharski et al., 1990). The posterior limb interconnects the orbitofrontal cortices, the inferior temporal lobes, and the amygdalas (Di Virgilio et al., 1999; Patel et al., 2010), regions known to be affected in schizophrenia (White et al., 2008a; Kanahara et al., 2013). Structural abnormalities have been reported in the AC of schizophrenia patients, including decreased WM density (Hulshoff Pol et al., 2004), reduced FA (Choi et al., 2011), and reduced fiber number (Highley et al., 1999). Reduced FA has also been reported in the AC of young bipolar patients (Saxena et al., 2012).

DTI studies in schizophrenia patients have also revealed alterations in other cortical and subcortical WM structures (Ellison-Wright and Bullmore, 2009; Segal et al., 2010; Abdul-Rahman et al., 2011; Lee et al., 2013; Canu et al., 2014; Holleran et al., 2014) that were not significantly altered in the brain of Gclm KO mice. While the identified FA reduction in FF and AC are the most prominent WM alterations resulting from a whole brain analysis in Gclm KO and WT mice, this does not exclude that other WM tracts may be slightly affected by deficiency in antioxidant systems. Interestingly, many of the WM tracts that could be analyzed in our study also displayed small and nonsignificant lower FA and higher RD in Gclm KO compared with WT mice. It should be noted that the small size of the mouse brain poses a challenge for detecting alterations in small or diffuse WM tracts. In particular, despite the high resolution achieved (voxel size was 0.16 x 0.31 x 0.6mm^3^), partial volume effects are prominent in cortico-cortical projections. In the CC, most studies in schizophrenia have found alterations in the genu, its most anterior part (Ellison-Wright and Bullmore, 2009). In our study, the CC ROI comprises all the antero-posterior range of the corpus callosum, which may have diluted possible alterations, but this lack of focal power in big ROIs is inherent to this exploratory study.

In conclusion, redox dysregulation caused by reduced glutathione synthesis has a negative impact on the structure and function of a subset of WM tracts. The alterations in FF and AC are present at peri-pubertal age and remain during adulthood without further worsening. These data suggest that redox dysregulation/oxidative stress could contribute to the impaired WM integrity found in schizophrenia and other psychiatric disorders.

## Statement of Interest

None.

## Supplementary Material

supplementary Figure 1
