# Supplementary material for: Glutathione Deficit Affects the Integrity and Function of the Fimbria/Fornix and Anterior Commissure in Mice: Relevance for Schizophrenia
Source: Int J Neuropsychopharmacol. 2015 Oct 3;19(3):pyv110. doi: 10.1093/ijnp/pyv110 (PMC4815475; doi:10.1093/ijnp/pyv110)
Supplement: supplementary Figure 1 [file pyv110supplemental_material.pdf]

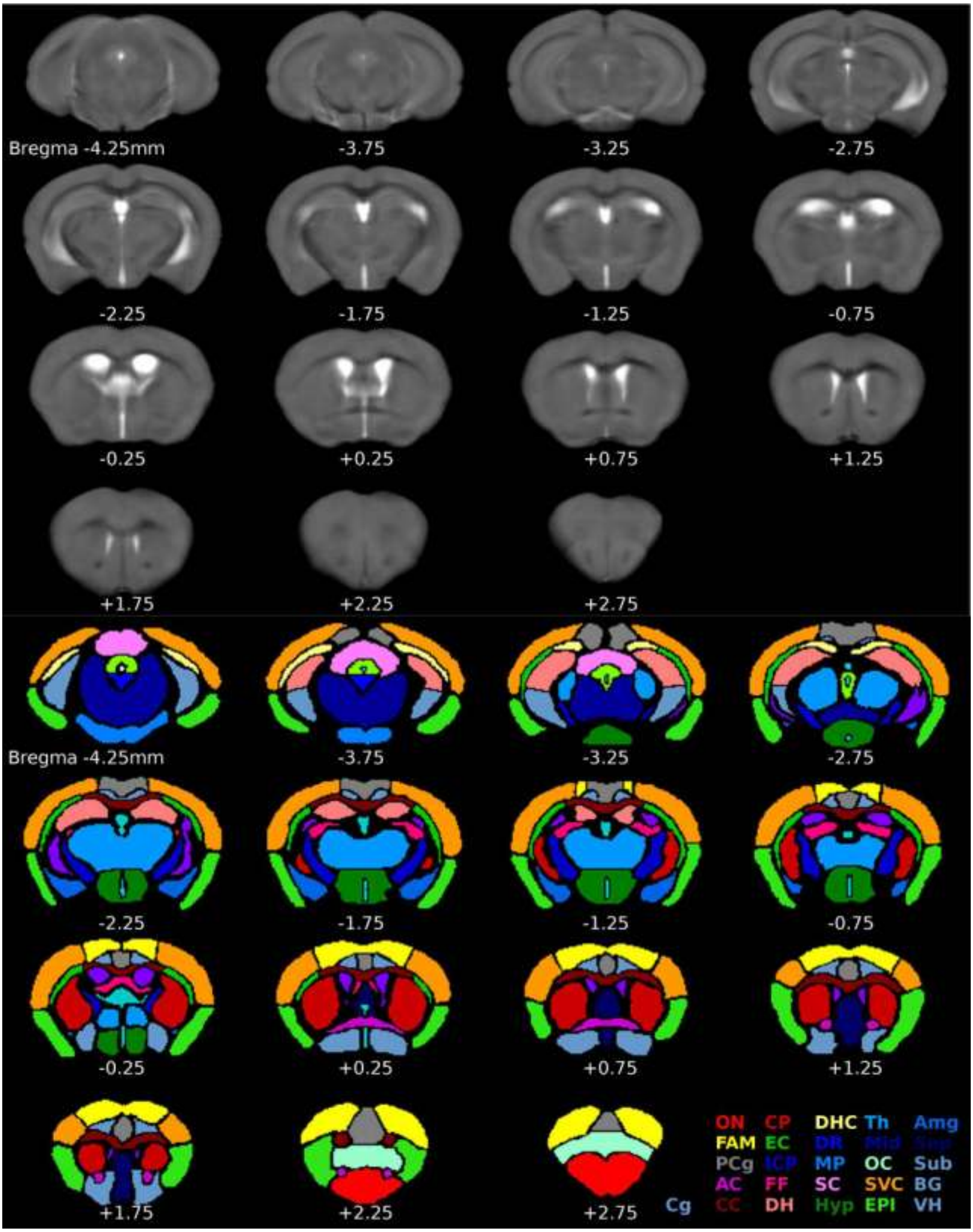

SUPPLEMENTAL MATERIAL

Figure 1. Region of interest (ROI) atlas used for the segmentation of the diffusion tensor imaging (DTI) data. The top panels show the T2.

Weighted average atlas used for registration. The bottom panels show the segmented ROIs. Coordinates from bregma are given under each slice. Color codes: olfactory nucleus (ON), frontal association and motor cortices (FAM), prelimbic and cingulate cortices (Pcg), anterior commissure (AC), corpus callosum (CC), caudate and putamen (CP), external capsule (EC), internal capsule and pallidum (ICP) fornix and fimbria (FF), dorsal hippocampus (DH), dorsal hippocampal commissure (DHC) dorsal raphe (DR), medulla and pons (MP), superior colliculi (SC), hypothalamus (Hyp), thalamus (Th), midbrain (Mid), orbital cortex (OC), sensory and visual cortex (SVC), entorhinal, piriform and insular cortex (EPI), amygdala and amygdaloid (Amg), septum (Sep), subiculum (Sub), basal ganglia (BG), ventral hippocampus (VH), cingulum (Cg).

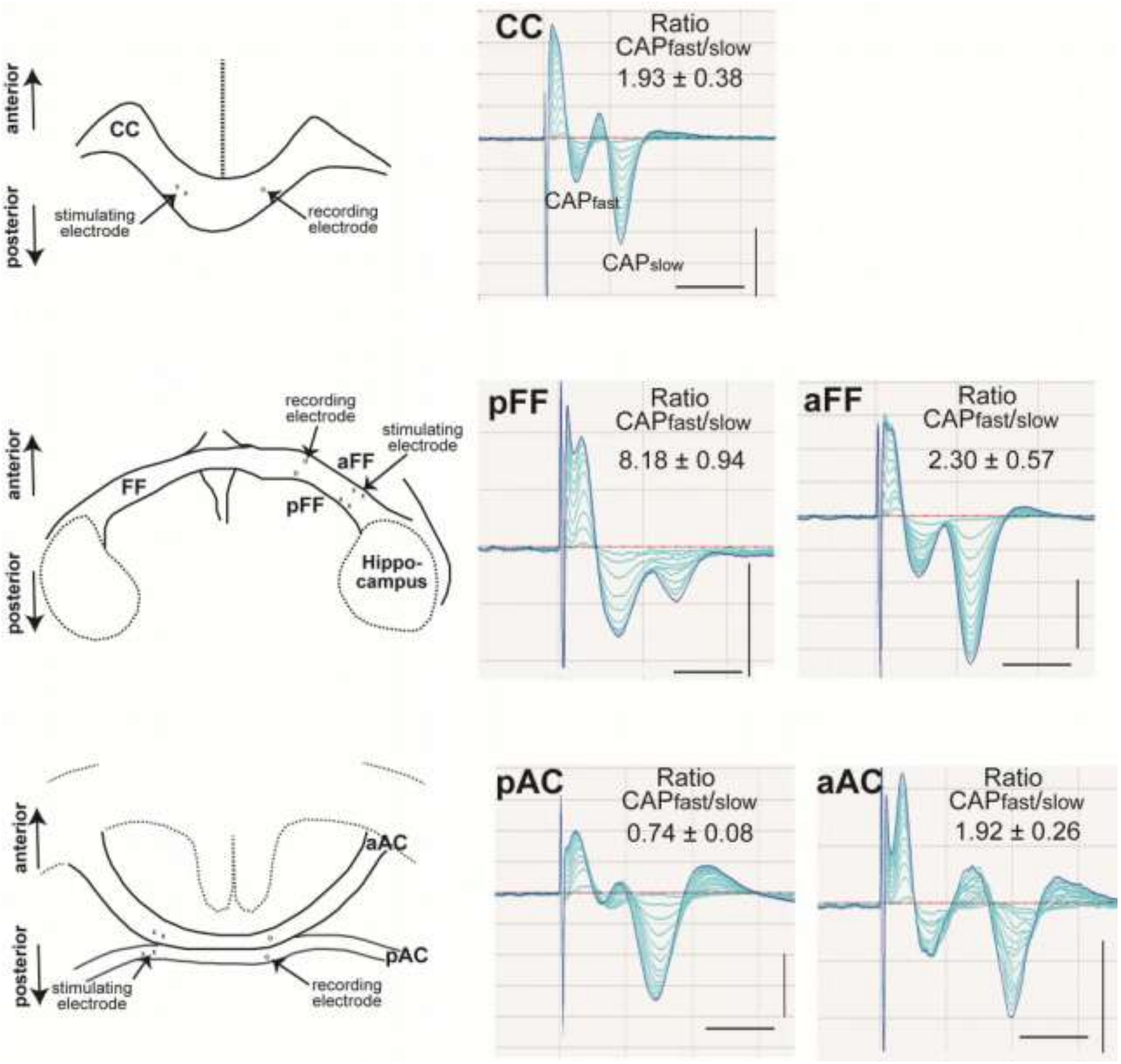

Figure 2. The drawings illustrate the placements of the stimulating (pair of small x) and recording (small dot) electrodes along, respectively, the corpus callosum (CC), the fornix-fimbria (FF), and the anterior commissure (AC). The traces are representative recordings of compound action potentials (CAPs) generated by electrical stimulations at increasing intensities along the CC, the posterior part of the FF (pFF), the anterior part of the FF (aFF), the posterior limb (pAC), and the anterior limb (aAC) of the AC in an adult wild-type mouse. It shows that the relative contribution of the CAPs from the fast-conducting (CAPfast) and slow-conducting fibers (CAPslow) differs in the different white matter (WM) tracts. It also shows that the amplitude of the CAP of the slow-conducting fibers is much smaller in the pFF than in the aFF. For illustration, the ratio between the amplitude of the CAPfast and that of the CAPslow is given for each WM

tract of wild-type mice (mean ± SEM, n = 8-11/WM tract). Horizontal bars: 2 ms; vertical bars: 1 mV.

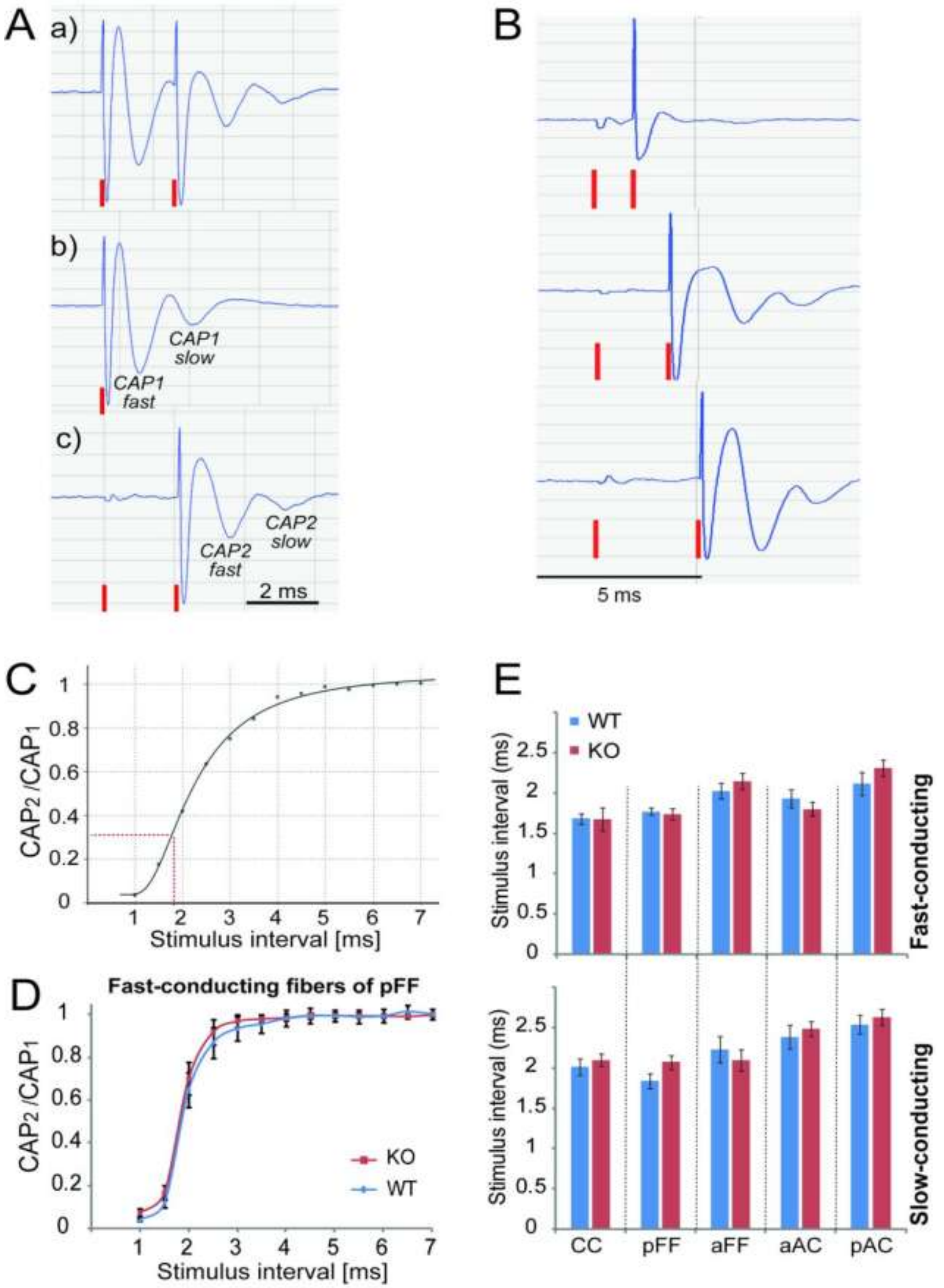

Figure 3. Refractory period in the fast- and slow-conducting fibers within the corpus callosum (CC), fornix-fimbria (FF), and the anterior commissure (AC) of adult *Gclm KO* and *WT* mice. The refractory period (time interval during which a second action potential cannot be initiated) can only be properly measured by recording a single fiber. In a fiber population, a generalized altered refractory period would affect how increasing the stimulus interval leads to an increase and recovery of the amplitude of the CAP evoked by the second stimulation. Therefore, the ratio between the CAP amplitude to the second stimulation (CAP2) and CAP amplitude to the first stimulation (CAP1) is plotted in function of stimulus interval. The stimulus interval that leads to a third of CAP2 was extrapolated based on a best fitted exponential equation applied to the data. The assumption was that the stimulus interval for which a third of CAP2 recovered would be longer in the case of a prolonged refractory period within a large proportion of fibers. (A) The raw trace a) shows an example of a response recorded in the CC of a *WT* mouse to a pair of stimuli presented 2 ms apart. From such a recording, it is not possible to properly measure the CAP amplitude of fast and slow-conducting fibers evoked respectively by the first and the second stimulation, as the responses to the 2 stimulations partially overlap. Therefore, CAP1 for fast- and slow-conducting fibers were estimated from the response to a single stimulation presented 10 s before the paired stimuli (see trace b). CAP2 for fast- and slow-conducting fibers were measured on trace c), which corresponds to the subtraction of trace b) from the raw trace a). (B) Example showing that CAP2 for fast- and slow-conducting fibers (in CC of a *WT* mouse) increases as the time interval between the first and second stimulation increases. Red bars depict the stimulations. (C) The ratio between the CAP2 and CAP1 is plotted in function of the stimulus interval (example from fast-conducting fibers in the CC of a *WT* mouse). Red dotted lines points to the stimulus interval that leads to a CAP2/CAP1 ratio of 0.3. (D) Example showing the plot of CAP2/CAP1 ratio in function of the stimulus interval for the fast-conducting fibers in the posterior part of FF (pFF) in *Gclm KO* and *WT* mice. Data are depicted by the mean ± SEM. (E) Stimulus interval corresponding to a CAP2/CAP1 ratio of 0.3 for the fast- and slow-conducting fibers of the CC, FF, and AC of adult *Gclm KO* and *WT* mice. No significant difference between genotype is observed,

suggesting no overall alteration of the refractory period in the fibers of the investigated WM tracts. Data are depicted by the mean ± SEM, n = 8 to 11/group. aAC, anterior limb of AC; aFF, anterior part of FF; pAC, posterior limb of AC.

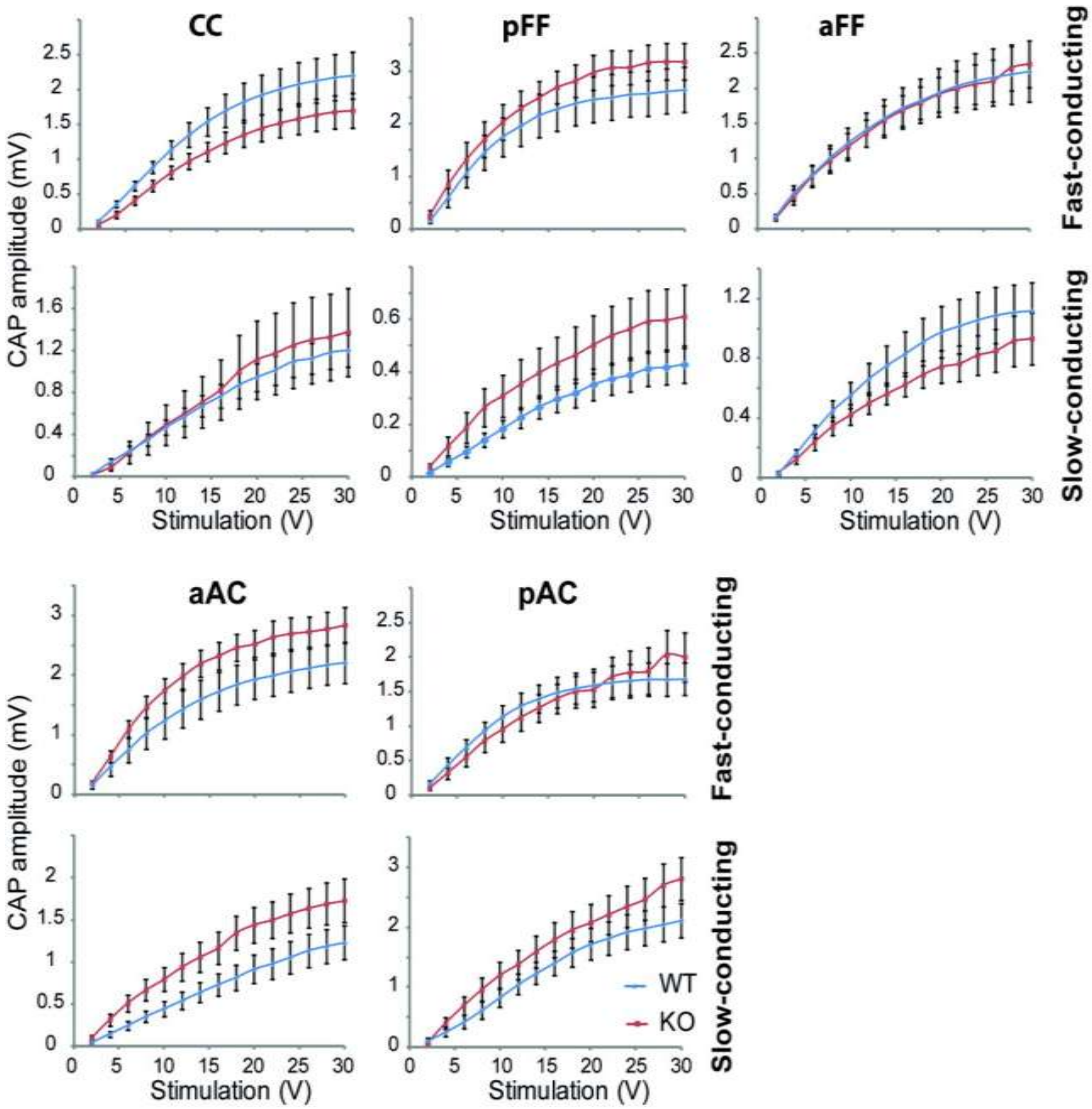

Figure 4. Relationship between absolute amplitude of compound action potentials (CAPs) and stimulation intensity for the fast- and slow-conducting fibers in the corpus callosum (CC), fornixfimbria (FF), and anterior commissure (AC) of adult *Gclm KO* and *WT* mice. Data are depicted by mean ± SEM (n =

8-11/group and stimulation intensity). aAC, anterior limb of AC; aFF, anterior part of FF; pAC, posterior limb of AC.

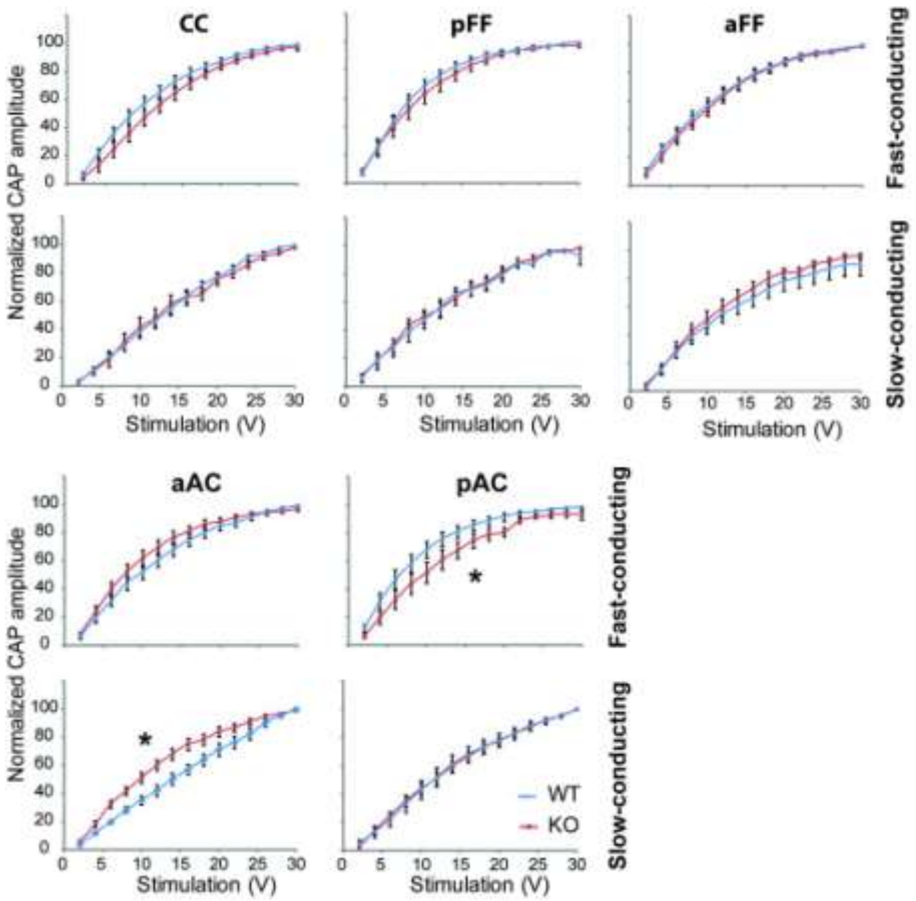

Figure 5. Relationship between normalized amplitude of compound action potentials (CAPs) and stimulation intensity for the fast- and slow-conducting fibers in the corpus callosum (CC), fornixfimbria (FF), and anterior commissure (AC) of adult *Gclm KO* and *WT* mice. The amplitude was normalized to the maximal response amplitude to render the response-dose independent of the number and density of excited fibers. Data are depicted by mean ± SEM. **P* < .05 Generalized Additive Mixed Model (n = 8 to 11/group and stimulation intensity). aAC, anterior limb of AC; aFF, anterior part of FF; pAC, posterior limb of AC.

Region of interest	Mean WT	Mean KO	WT-KO (%)	WT-KO SD (%)	p	p corrected
Fornix and Fimbria	0.63	0.67	-6.6	1.7	0.001	*0.014
Internal Capsule and Pallidum	0.57	0.58	-3.0	1.3	0.022	0.595
Dorsal Hippocampal Commissure	0.54	0.56	-3.2	1.0	0.003	0.087
Medulla and Pons	0.60	0.61	-1.0	3.1	0.739	1
Corpus Callosum	0.58	0.59	-2.5	1.2	0.038	1
Anterior Commissure	0.61	0.63	-4.8	1.9	0.013	0.345
Septum	0.66	0.68	-2.8	1.7	0.102	1
Cingulum	0.59	0.60	-0.5	0.6	0.419	1
Midbrain	0.61	0.62	-1.7	1.7	0.322	1
External Capsule	0.62	0.64	-3.5	1.7	0.042	1
Thalamus	0.62	0.64	-2.0	1.2	0.094	1
Basal Ganglia	0.62	0.64	-2.1	2.0	0.283	1
Superior Colliculi	0.64	0.65	-1.3	0.9	0.136	1
Subiculum	0.63	0.64	-1.3	1.9	0.493	1
Hypothalamus	0.70	0.72	-2.8	1.7	0.096	1
Amygdala and Amygdaloid	0.63	0.65	-3.0	1.7	0.083	1
Olfactory Nucleus	0.69	0.69	0.5	1.8	0.778	1
Dorsal Raphe	0.67	0.69	-2.6	2.0	0.183	1
Entorhinal, Piriform and Insular Cortex	0.65	0.66	-1.9	1.3	0.146	1
Orbital Cortex	0.63	0.64	-2.0	1.2	0.089	1
Periaqueductal Gray	0.68	0.69	-1.1	1.3	0.401	1
Frontal Association and Motor Cortex	0.62	0.62	-1.2	0.6	0.038	1
Caudate and Putamen	0.62	0.63	-1.6	1.1	0.114	1
Prelimbic and Cingulate Cortex	0.63	0.64	-1.3	0.5	0.014	0.39
Sensory and Visual Cortex	0.63	0.63	-1.3	0.7	0.045	1
Dorsal Hippocampus	0.65	0.66	-2.6	0.9	0.007	0.183
Ventral Hippocampus	0.69	0.72	-4.8	1.9	0.014	0.386

Table S1: Mean RD values per ROI and genotype in mm^2/s $x10^3$, difference between genotypes and standard error of the difference estimated by a mixed-effects linear model using genotype and age as fixed factors and intercepts for each subject as random factor. The first and second columns are calculated by averaging the values of the three ages for each animal and then the average of all animals of the respective genotype. The third column is calculated as (mean *WT* – mean *KO*) x 100 / mean *WT*. The fourth column shows the standard deviation of the difference (mean *WT* – mean *KO*) also in percentage of mean *WT*. P-values for genotype differences from the likelihood ratio tests before and after correction for multiple comparisons are given in the fifth and sixth columns respectively.

Region of interest	Mean WT	Mean KO	WT-KO (%)	WT-KO SD (%)	p	p corrected
Fornix and Fimbria	1.29	1.32	-2.2	1.4	0.128	1
Internal Capsule and Pallidum	1.08	1.10	-1.9	1.1	0.083	1
Dorsal Hippocampal Commissure	1.04	1.05	-0.5	0.9	0.571	1
Medulla and Pons	1.10	1.07	2.3	2.0	0.253	1
Corpus Callosum	1.01	1.03	-2.2	1.1	0.048	1
Anterior Commissure	0.96	0.97	-0.6	1.4	0.646	1
Septum	1.04	1.09	-4.2	1.4	0.005	0.129
Cingulum	0.89	0.90	-1.7	0.6	0.005	0.144
Midbrain	0.93	0.96	-2.8	1.4	0.042	1
External Capsule	0.90	0.93	-2.9	1.5	0.063	1
Thalamus	0.88	0.89	-1.8	1.0	0.065	1
Basal Ganglia	0.88	0.91	-2.5	1.6	0.104	1
Superior Colliculi	0.90	0.91	-1.3	1.0	0.185	1
Subiculum	0.87	0.90	-3.1	1.6	0.048	1
Hypothalamus	0.97	0.98	-1.5	1.3	0.248	1
Amygdala and Amygdaloid	0.88	0.91	-3.0	1.4	0.032	0.874
Olfactory Nucleus	0.95	0.96	-1.1	1.5	0.472	1
Dorsal Raphe	0.92	0.96	-4.7	1.8	0.01	0.259
Entorhinal, Piriform and Insular Cortex	0.88	0.90	-2.3	1.2	0.057	1
Orbital Cortex	0.85	0.88	-3.4	1.1	0.002	0.059
Periaqueductal Gray	0.93	0.96	-3.0	1.4	0.033	0.884
Frontal Association and Motor Cortex	0.81	0.82	-1.5	0.6	0.009	0.248
Caudate and Putamen	0.81	0.83	-2.3	1.0	0.019	0.526
Prelimbic and Cingulate Cortex	0.80	0.82	-1.6	0.6	0.009	0.243
Sensory and Visual Cortex	0.80	0.82	-2.3	0.8	0.005	0.125
Dorsal Hippocampus	0.82	0.84	-2.7	1.0	0.011	0.302
Ventral Hippocampus	0.84	0.88	-5.2	2.1	0.014	0.371

Table S2: Mean AD values per ROI and genotype in mm^2/s $x10^3$, difference between genotypes and standard error of the difference estimated by a mixed-effects linear model using genotype and age as fixed factors and intercepts for each subject as random factor. The first and second columns are calculated by averaging the values of the three ages for each animal and then the average of all animals of the respective genotype. The third column is calculated as (mean *WT* – mean *KO*) x 100 / mean *WT*. The fourth column shows the standard deviation of the difference (mean *WT* – mean *KO*) also in percentage of mean *WT*. P-values for genotype differences from the likelihood ratio tests before and after correction for multiple comparisons are given in the fifth and sixth columns respectively.

Region of interest	Mean WT	Mean KO	WT-KO (%)	WT-KO SD (%)	p	p corrected
Fornix and Fimbria	0.85	0.89	-4.4	1.5	0.005	0.13
Internal Capsule and Pallidum	0.74	0.76	-2.5	1.2	0.035	0.942
Dorsal Hippocampal Commissure	0.71	0.72	-1.9	0.8	0.018	0.499
Medulla and Pons	0.77	0.76	0.6	2.4	0.793	1
Corpus Callosum	0.72	0.74	-2.4	1.0	0.022	0.591
Anterior Commissure	0.72	0.75	-2.9	1.6	0.065	1
Septum	0.79	0.82	-3.4	1.5	0.027	0.72
Cingulum	0.69	0.70	-1.0	0.5	0.059	1
Midbrain	0.72	0.74	-2.2	1.6	0.158	1
External Capsule	0.71	0.74	-3.2	1.6	0.048	1
Thalamus	0.71	0.72	-1.9	1.1	0.08	1
Basal Ganglia	0.71	0.73	-2.3	1.8	0.197	1
Superior Colliculi	0.73	0.74	-1.2	0.8	0.12	1
Subiculum	0.71	0.72	-2.1	1.7	0.215	1
Hypothalamus	0.79	0.81	-2.3	1.5	0.127	1
Amygdala and Amygdaloid	0.72	0.74	-3.0	1.6	0.053	1
Olfactory Nucleus	0.78	0.78	-0.2	1.7	0.924	1
Dorsal Raphe	0.75	0.78	-3.4	1.8	0.057	1
Entorhinal, Piriform and Insular Cortex	0.73	0.74	-2.1	1.3	0.1	1
Orbital Cortex	0.70	0.72	-2.6	1.1	0.021	0.564
Periaqueductal Gray	0.77	0.78	-1.8	1.3	0.144	1
Frontal Association and Motor Cortex	0.68	0.69	-1.3	0.5	0.016	0.421
Caudate and Putamen	0.68	0.70	-1.9	1.0	0.06	1
Prelimbic and Cingulate Cortex	0.69	0.70	-1.4	0.5	0.009	0.239
Sensory and Visual Cortex	0.68	0.69	-1.7	0.7	0.012	0.328
Dorsal Hippocampus	0.70	0.72	-2.6	0.9	0.008	0.204
Ventral Hippocampus	0.74	0.77	-5.0	1.9	0.013	0.345

Table S3: Mean MD values per ROI and genotype in mm^2/s x10^3, difference between genotypes and standard error of the difference estimated by a mixed-effects linear model using genotype and age as fixed factors and intercepts for each subject as random factor. The first and second columns are calculated by averaging the values of the three ages for each animal and then the average of all animals of the respective genotype. The third column is calculated as (mean *WT* – mean *KO*) x 100 / mean *WT*. The fourth column shows the standard deviation of the difference (mean *WT* – mean *KO*) also in percentage of mean *WT*. P-values for genotype differences from the likelihood ratio tests before and after correction for multiple comparisons are given in the fifth and sixth columns respectively.
